# Obituary

**Published:** 1852-12

**Authors:** 


*Obituary.* — The sudden death of that distinguished writer and teacher, the late Prof. Daniel Drake, cannot but produce a profound sensation throughout our country, but more especially in the southern and western sections of the republic. Dr. Drake has long been identified with medical education and literature in the south-west. He was for many years connected with the Transylvania Medical School, at Lexington, in the palmy days of that institution. The Ohio Medical College, at Cincinnati, was founded mainly by his exertions. With the Louisville Med. College he had been connected for a long period, having resigned his professorship last summer to resume a chair in his favorite school at Cincinnati. He lectured for one or more sessions, many years ago, at the Jefferson Med. School at Philadelphia.

For a long period he was associated in the editorial management of the Western Journal of Medicine and Surgery.

In the spring of 1850, the first volume of his great work on the climate, topography, diseases, etc., of the interior valley, was published. In collecting materials for this work he had been engaged for twenty years. He had expected to have entered on the publication of the second volume early in the next spring. It is much to be hoped that the preparation of the remainder of the work is so far advanced, that it will not be left, by his death, incomplete.

Dr. Drake was endowed with a vigorous intellect, and possessed rare qualities as a public teacher. All who have listened to his lectures speak in high terms of the learning and thought which they embodied, and of the power and often eloquence with which they were delivered. He was a laborious student, and had sufficient strength of mind to resist that weakness, unfortunately too often incident to advancing age, which leads to a neglect and undervaluing of the new developments marking the advancement of medical science. Although Dr. Drake, from his years, might be entitled to be ranked among the adherents to "*old physic*," his sympathies were equally with the younger and progressive school. He did not fetter his mind with an unchangeable adherence to solidism, or any other scientific dogma, but was ever ready to welcome truth albeit it demanded the sacrifice of opinions perhaps long cherished. He was a philosopher in the true sense of that term, not a partisan. Truth was his aim and ruling motive, not a pride of opinion or of consistency.

Dr. Drake died a few days after reaching the age of sixty-seven. His appearance did not denote so many years. We had the pleasure of meeting him but a few days before his death, and of hearing one of his last public efforts. He was present at the supper given by the physicians of Louisville to the members of the Kentucky State Medical Society, and in a brief speech on that occasion, entertained his hearers with a historical account of medical education in the south-west based on his own reminiscences. At that time he appeared to be in vigorous health, and there seemed every reason for hoping that many years more of labor and usefulness might be added to the past. On his return to Cincinnati, however, he was attacked with congestion of the brain, an affection to which he had been occasionally subject. It eventuated in coma, and death after an illness of a few days.

The circumstances of his death were those which he had frequently said he should prefer should accompany that event. He often expressed to his friends a desire to die in the midst of his labors as a teacher, and that his remains should be followed to the grave by the students whom he was accustomed to meet in the lecture room. In these respects it pleased Providence to conform to his wishes. He died shortly after the opening of the session of the Medical College of Ohio, and the members of the class there assembled were among the mourners at the funeral.

Dr. Drake for many years before his death was a professing Christian, being a member of the Protestant Episcopal church.

